# Prevalence of Microbiologically Confirmed Female Genital Tuberculosis in a Tertiary Care Hospital in Faridabad, Haryana, India

**DOI:** 10.7759/cureus.75358

**Published:** 2024-12-09

**Authors:** Jayanthi Gunasekaran, Rahul Ranjan, Priya Karlapudi, Pooja Pandey, Karteeka Chichhal, Sonam Gupta, Ankita Rai, Ina Joshi, Umesh Kumar, Rajiv M Gupta

**Affiliations:** 1 Department of Microbiology, ESIC Medical College and Hospital, Faridabad, IND

**Keywords:** cbnaat, endometrial biopsy, extrapulmonary tb, genital tb, infertility, lowenstein jensen culture, molecular testing, mycobacterium tuberculosis, paucibacillary, ziehl-neelsen staining

## Abstract

Aim: This study aimed to determine the prevalence of microbiologically confirmed female genital tuberculosis (FGTB) infection in patients attending a tertiary care hospital in North India.

Materials and methods: A total of 623 endometrial biopsy samples were processed in the mycobacteriology laboratory from the outpatient and inpatient gynecology departments between May 2022 and February 2024. Ziehl-Neelsen (ZN) smear was performed on all samples. Each sample was subsequently subjected to cartridge-based nucleic acid amplification test (CBNAAT) Xpert *Mycobacterium tuberculosis *(MTB)/rifampicin (RIF) Ultra (Cepheid, Sunnyvale, California), and LJ culture irrespective of the ZN smear result and demographic details of the patients were recorded simultaneously.

Results: Of the 623 endometrial biopsy samples tested, 17 (2.73%) samples were positive for MTB by CBNAAT Ultra. The disease was most commonly observed in the reproductive age group between 21 and 35 years. ZN smear and culture were negative for all the samples received for endometrial TB. Of these 17 positive samples, one (5.9 %) was MTB detected (medium), four (23.5 %) (low), two (11.8 %) (very low), and 10 (58.8 %) (trace). RIF resistance was detected in one (5.9 %) of these 17 samples. However, the 10 samples that were detected as MTB trace and RIF indeterminate ideally need to be processed further by culture or by line probe assay to determine the drug resistance.

Conclusion: Genital TB is characterized by a low bacterial load. Therefore, a highly sensitive and specific molecular test is essential for confirming the diagnosis microbiologically. Our study found that Xpert MTB/RIF Ultra is particularly valuable because it detected 58.8% of cases as “TRACE,” which might be missed by other testing methods. Early diagnosis using this test can significantly impact patient management, especially in cases related to infertility. This study effectively highlights the role of GeneXpert Ultra in documenting extra-pulmonary TB, including genital TB.

## Introduction

In 2022, a record 7.5 million people worldwide were newly diagnosed with tuberculosis (TB), marking the highest number since the World Health Organization began global TB monitoring in 1995 [1]. India recorded 2.8 million new TB cases, accounting for over a quarter of the world’s total [2]. Tragically, during that year, an estimated 342,000 deaths occurred due to TB in India, equivalent to 39 citizens losing their lives to this disease every hour [2].

Extrapulmonary TB (EPTB) constitutes 15% of all newly diagnosed TB cases. It commonly affects the lymph nodes, pleura, peritoneum, bones and joints, genitourinary system, and meninges [3].

Female genital tuberculosis (FGTB) is primarily caused by *Mycobacterium tuberculosis* (MTB), although it can occasionally be due to *Mycobacterium bovis* or atypical mycobacteria. This condition typically arises as a secondary infection following TB in the lungs or other organs. The infection can spread to the genital tract through the bloodstream (hematogenous route), lymphatic system, or direct extension from abdominal TB [4]. The incidence of FGTB, a form of EPTB, is on the rise among young women worldwide [5,6]. FGTB can lead to menstrual irregularities and infertility in women [6,7]. Early detection and appropriate combination treatment regimens with adequate drug dosages can help minimize damage and prevent future infertility in affected women. Therefore, this study was designed to profile the prevalence of FGTB and rifampicin resistance patterns in microbiologically confirmed FGTB cases at a tertiary care hospital in India using genotypic methods.

## Materials and methods

Aims and objectives

This study aimed to assess the prevalence of microbiologically confirmed FGTB in patients presenting with symptoms and signs of genital TB at ESIC Medical College and Hospital (ESIC MC&H), Faridabad, a tertiary care hospital in North India. The study objectives were to investigate the role of GeneXpert Ultra in detecting FGTB, to assess the MTB load in positive cases, and to identify rifampicin (RIF) resistance in confirmed FGTB cases. In addition, it sought to determine the age distribution of patients affected by genital TB.

The Institutional Ethics Committee of ESIC MC&H, Faridabad, issued approval (file no. 34 X/11/13/2023- IEC/DHR/92).

Study population

This prospective study was conducted in the Department of Microbiology of ESIC MC&H, Faridabad, from May 2022 to February 2024. *Inclusion Criteria*

Endometrial biopsy samples were sent to the microbiology laboratory for testing from clinically suspected patients of FGTB from both the outpatient and inpatient gynecology departments. *Exclusion Criteria*

All the pulmonary (i.e., sputum, endotracheal secretions, bronchoalveolar lavage (BAL), etc.) and extrapulmonary samples from patients other than clinically suspected patients of FGTB were excluded.

Sample size

Convenient sampling was done, and 623 endometrial biopsy samples collected over a period of 22 months were included in the study.

Sample processing

Ziehl-Neelsen (ZN) smear was performed on all samples. Each of the samples was subsequently subjected to CBNAAT (Xpert MTB/RIF Ultra) (Cepheid, Sunnyvale, California) and LJ culture irrespective of the ZN smear result.

Data collection

Patient demographic details were recorded using the patient requisition form after obtaining consent. Details like name, age, sex, residential address, and date of registration, as well as laboratory details such as the date of sample collection, date of sample processing, date of reporting, type of sample, and test results, were recorded.

The CBNAAT - Xpert MTB/RIF Ultra results were documented as either theMTBcomplex detected or not detected. This was followed by noting rifampicin resistance as detected, not detected, or indeterminate, and the MTB complex load was categorized as high, medium, low, very low, or trace.

Data analysis

All collected details were documented in Excel (Microsoft Corporation, Redmond, Washington), and the results were analyzed based on the objectives.

## Results

Of the 623 endometrial biopsy samples tested, 17 (2.73%) samples were positive for MTB by CBNAAT Ultra. ZN smear and LJ culture were negative for all the samples received for endometrial TB. The disease was most commonly observed in the age group between 21 and 35 years depicted in Table 1.

**Table 1:** Age group distribution of patients tested for FGTB that were confirmed MTB positive FGTB: female genital tuberculosis, MTB: *Mycobacterium tuberculosis*

Age (Years)	Number (%)
15-20	1/17 (5.9 %)
21-25	6/17 (35.3 %)
26-30	5/17 (29.4 %)
31-35	4/17 (23.5 %)
36-40	1/17 (5.9 %)

CBNAAT - Xpert MTB/RIF Ultra in addition to the detection of the MTB complex, and RIF resistance also detects the mycobacterial load as high, medium, low, very low, and trace. Of the 17 positive samples, one (5.9 %) was MTB detected (medium), four (23.5 %) (low), two (11.8 %) (very low), and 10 (58.8 %) (trace), as depicted in Table 2.

**Table 2:** Mycobacterium tuberculosis complex load by CBNAAT - Xpert MTB/RIF Ultra CBNAAT: cartridge-based nucleic acid amplification test, MTB: Mycobacterium tuberculosis, RIF: rifampicin

MTB load	Number (%)
High	0/17 (0 %)
Medium	1/17 (5.9 %)
Low	4/17 (23.5 %)
Very low	2/17 (11.8 %)
Trace	10/17 (58.8 %)

The CBNAAT - Xpert MTB/RIF Ultra test also evaluates RIF resistance in MTB-positive samples, categorizing results as detected, not detected, or indeterminate. Among the 17 MTB-positive samples, RIF resistance was detected in only one sample (5.9%). In 10 samples (58.8%) with a trace MTB load, RIF resistance was indeterminate. When RIF resistance results are indeterminate, it is recommended to retest the sample using the same or a different molecular test to confirm the result. If the repeat test is also indeterminate, phenotypic drug susceptibility testing (DST) should be performed to determine RIF resistance. However, we could not perform repeat testing in our study.

## Discussion

Genitourinary disease is the most prevalent form of EPTB, representing 27% of cases globally, with a range of 14% to 41% [8].

The prevalence of FGTB in India is quite high. According to a survey by the Indian Council of Medical Research (ICMR), it increased from 19% in 2011 to 30% in 2015 [6]. This indicates a significant public health concern, especially considering its impact on female fertility. FGTB typically arises as a secondary condition following pulmonary TB, spreading through the bloodstream. It can also be disseminated via the lymphatic system or directly from abdominal TB. Although rare, FGTB can develop after sexual contact with a man who has TB of the penis or epididymis. This condition is more prevalent in premenopausal women, possibly because the atrophic endometrium in postmenopausal women creates an unfavorable environment for mycobacterial growth [9].

FGTB predominantly affects the fallopian tubes, with 90-100% of cases presenting lesions such as endo salpingitis, exo salpingitis, interstitial salpingitis, or salpingitis isthmica nodosa. The uterine endometrium is involved in 50-80% of cases, showing lesions like ulcers, synechiae, and adhesions. Ovarian involvement occurs in 20-30% of cases, also presenting with ulcers, synechiae, and adhesions. The cervix is affected in 5-15% of cases, with lesions including polypoidal growth and ulceration. The vulva and vagina are rarely involved, seen in only 1% of cases, and may present with growths, ulceration, and fistulas [9].

In around 10% of cases, FGTB may be asymptomatic [4]. Patients may exhibit fever and constitutional symptoms like malaise, anorexia, and weight loss. However, not all patients manifest these symptoms. The most common presentation is infertility (either primary or secondary). In addition, patients frequently experience menstrual disturbances, which can be longstanding and varied in their manifestations. Early symptoms may include heavy menstrual bleeding. Patients may also present with dysmenorrhea, puberty menorrhagia, or postmenopausal bleeding. Other complaints include oligomenorrhea, hypomenorrhea, and amenorrhea. Some patients may report chronic abdominal or pelvic pain, and a palpable mass may be detected during clinical examination. A subset of patients may also describe vaginal discharge of varying duration and quality, while others may experience urinary disturbances such as incontinence [10].

FGTB is widely recognized as a significant etiological factor for infertility in regions with a high prevalence of TB. This disease not only causes tubal obstruction and dysfunction but also impairs implantation due to endometrial involvement and leads to ovulatory failure from ovarian involvement [9]. The burden of TB is often underestimated because many patients are asymptomatic and are typically diagnosed only during infertility evaluations [9]. According to the 2016 Index TB guidelines (guidelines on EPTB) [11], the diagnosis of FGTB can be established based on any of the following criteria: typical laparoscopic appearance suggestive of FGTB (such as tubercles, caseation, or beaded tubes), detection of acid-fast bacilli (AFB) in any gynecological specimen using microscopy or GeneXpert, positive culture results for MTB from any gynecological sample, histopathological examination showing findings consistent with FGTB.

The prevalence of CBNAAT-confirmed genital TB in our study was determined to be 2.73%. Zahoor D et al. [9] reported a prevalence of 7.25% (five positives by GeneXpert out of 69 samples tested) in their study. Keshari et al. [8] reported a prevalence of 7.84 % in their study. In a study conducted by Sharma et al. [12], 68 women underwent diagnostic laparoscopy, and among them, six (8.82%) tested positive for MTB using CBNAAT, while 10 (5%) were positive among a total of 200 suspected cases of MTB in their study using CBNAAT. In a study conducted by Tiwari K et al. [13], GeneXpert yielded positive results in only two out of 176 women tested, representing 1.14% of the samples. The true prevalence of genital TB in our setting is likely higher than reported, as many cases are asymptomatic or present with non-specific symptoms, leading to potential underdiagnosis. In cases of FGTB involving sites such as the fallopian tubes and ovaries, obtaining samples for microbiological testing to detect MTB can be challenging. Consequently, there is a possibility that cases of genital TB may have been overlooked as we did not include laparoscopic findings in our study. In our study, the ZN smear was negative for all samples received for endometrial TB. Similarly, in the study by Zahoor D et al. [9], none of the 193 samples submitted to the lab tested positive for AFB microscopy. This could be attributed to the paucibacillary nature of the specimens. In a study by Sharma D et al. [12], 0.5% (one out of 200) of patients suspected to have MTB were confirmed positive for MTB using the ZN smear technique.

In our study, LJ culture was negative for all samples. However, the culture for MTB was positive in 5.18% (10 of the 193 samples tested) in the study by Zahoor D et al. [9]. In a study conducted by Sharma and colleagues [12], 2.94% (two out of 68) patients who underwent diagnostic laparoscopy tested positive for MTB using LJ culture, while four (2%) were positive among a total of 200 suspected cases of MTB in their study. Tiwari K et al. [13] reported a culture positivity of 1.7% (three of the 176 samples) in their study. According to the Index TB guidelines (2016) [11], the recommended treatment for cases of FGTB consists of the following regimen: The initial phase (two months) includes HRZE (isoniazid (H), rifampicin (R), pyrazinamide (Z), and ethambutol (E), while the continuation phase (four months) includes HRE (isoniazid (H), rifampicin (R), and ethambutol (E)).

The strength of our study lies in the extensive testing of 623 samples from patients suspected of having FGTB. This is the first study to examine such a large sample size using the CBNAAT - Xpert MTB/RIF Ultra assay. The GeneXpert Ultra assay has demonstrated notable performance metrics in diagnosing TB. Specifically, it shows a sensitivity of 83.7% and a specificity of 63.8%. The positive predictive value (PPV) is 20.4% and its negative predictive value (NPV) is impressive at 97.2% [14]. The assay system features two distinct multicopy amplification targets, IS6110 and IS1081, and boasts a larger DNA reaction chamber, expanding from 25 μL in GeneXpert to 50 μL in XpertUltra. In addition, XpertUltra includes comprehensive nucleic acid amplification capabilities and a more rapid thermal cycle, allowing it to detect as low as 16 CFU/mL, a significant improvement over GeneXpert’s 114 CFU/mL detection limit [15]. The assay also categorizes the MTB load as high, medium, low, very low, or trace, which demonstrated the paucibacillary nature of most of the specimens that tested positive for MTB in our study. Among the samples that tested positive for MTB, 58.82% were classified as "trace." This finding is particularly noteworthy, as these cases might be overlooked when tested with less sensitive methods. Our study has certain limitations. As our study was conducted in a laboratory setting, the gravida status of the female presented with infertility in this study was unknown. We did not analyze the clinical details of the patient. We did not evaluate treatment follow-up and outcome of patients. We did not analyze the patients who were clinically diagnosed with genital TB based on laparoscopic appearance.

## Conclusions

FGTB is often underestimated due to its asymptomatic nature. Also, FGTB is characterized by a low bacterial load, necessitating a highly sensitive and specific molecular test for microbiological confirmation. Our study found that the Xpert MTB/RIF Ultra test is particularly valuable, detecting 58.82% of cases as "trace," which might be missed by other testing methods. Early diagnosis using this test can significantly impact patient management, especially in infertility cases. This study effectively highlights the role of GeneXpert Ultra in documenting EPTB, including genital TB.
